# Neurocomputational Nosology: Malfunctions of Models and Mechanisms

**DOI:** 10.3389/fpsyg.2016.00602

**Published:** 2016-05-03

**Authors:** David L. Barack, Michael L. Platt

**Affiliations:** ^1^Departments of Philosophy, Neuroscience, and Economics, Center for Science and Society, Columbia University in the City of New YorkNew York, NY, USA; ^2^Department of Philosophy, Duke UniversityDurham, NC, USA; ^3^Duke Institute for Brain Sciences, Duke UniversityDurham, NC, USA; ^4^Departments of Neurobiology and Psychology and Neuroscience, Duke UniversityDurham, NC, USA; ^5^Departments of Neuroscience, Psychology, and Marketing, University of PennsylvaniaPhiladelphia, PA, USA

**Keywords:** decision-making, computational psychiatry, neuropsychology, executive function, wisconsin card sorting test, Iowa Gambling Task, medial prefrontal cortex (mPFC)

## Abstract

Executive dysfunctions, psychopathologies arising from problems in the control and regulation of behavior, can occur as a result of the faulty execution of formal information processing models or as a result of malfunctioning neural mechanisms. The models correspond to the formal descriptions of how signals in the environment must be transformed in order to behave adaptively, and the mechanisms correspond to the signal transformations that nervous systems implement in order to execute those cognitive functions. Mechanisms in the form of repeated patterns of neural dynamics execute information processing models. Two distinct modes of malfunction can occur when neural dynamics execute models of information processing. The processing models describing behavior may fail to be executed correctly by neural mechanisms. Or, the neural mechanisms may malfunction, failing to implement the right computation. As an example of malfunctioning models in executive cognition, purported failures of rule following can be understood as failures to appropriately execute a suite of processing models. As an example of malfunctioning mechanisms of executive cognition, maladaptive behavior resulting from dysfunction in the medial prefrontal cortex (mPFC) can be understood as failures in the signal transformations carried out therein. The purpose of these examples is to illustrate the potential benefits of considering models and mechanisms in the diagnosis and etiology of neuropsychological illness and dysfunction, especially disorders of executive cognition.

## Introduction

In 1967, a subway train tragically struck Vladimir, a Muscovite engineering student, resulting in the resection of his frontal poles (Goldberg, 2001). Vladimir exhibited a range of deficits in complex behavior. When instructed to repeat a story, he was unable to recapitulate the correct narrative, as well as being unable to stop the rambling, incoherent narrative he did produce. When instructed to draw a circle, he drew multiple; when instructed to draw a cross, a circle, and a square in sequence, the cross appeared in all three shapes; when instructed to draw a circle, a cross, and a circle in sequence, Vladimir drew a donut last, persevering in drawing two lines as he had done for the cross. The annals of neurology are replete with examples of deficits in complex behavior such as Vladimir's.

Tempting though it may be to describe Vladimir as inappropriately persisting in following a rule, the accurate description and diagnosis of his dysfunctional psychology is an open scientific question. Understanding the information processing models and dynamical mechanisms of cognition is critical to understanding and diagnosing mental illness, including maladaptive perseverative behavior like Vladimir's. Here we discuss how models and mechanisms can provide new insight into psychiatric nosology. In particular, psychiatric pathology results from misexecution of processing models by dynamical mechanisms in the brain. We illustrate this approach by examining how a model-based view interprets pathology evident in rule-following tasks and how a mechanism-based view interprets pathology evident in medial prefrontal cortex (mPFC) dysfunction.

The models describe how signals in the environment must be transformed in order to behave adaptively, and the mechanisms describe the dynamics of neural circuits that execute those cognitive functions (Marr, 1982; Adams et al., 2012). Neuroscientists, psychologists, philosophers, and others who study the mind invoke formal, mathematical models of behavior to characterize cognitive functions. These models are mathematical formulae that contain variables, picking out which properties of the environment the system must track, and the mathematical relations between those variables, how they must be transformed, for adaptive behavior. For example, in the case of simple decisions, such as deciding between different size rewards or deciding when to leave a depleting resource, the models can be drawn from economic models of maximization of value (Bernoulli, 1738; Platt and Glimcher, 1999) or from optimal foraging theory (Charnov, 1976; Stephens and Krebs, 1986). The dynamics of the neural mechanisms, the neurons or neural circuits, execute these processing models by implementing neural computations over signals (Herz et al., 2006; Adams et al., 2012; Carandini and Heeger, 2012). These circuits implement these computations by transforming afferent signals they receive into efferent signals passed on to later processing areas. One example is the integrate-to-bound dynamical system, a neurocomputation for integrating noisy perceptual evidence during decision-making (Gold and Shadlen, 2001, 2007; Roitman and Shadlen, 2002) as well as potentially playing a role in strategic decisions to depart depleting resources (Hayden et al., 2011). Another example is divisive normalization, a neurocomputation involved in such diverse cognitive functions as attention (Reynolds and Heeger, 2009), value encoding (Louie et al., 2011), multisensory integration (Ohshiro et al., 2011), and others. The discovery that neural mechanisms repeatedly use a discrete number of neurocomputations, concatenated and combined to execute processing models, is a hallmark of recent advances in understanding the neural basis of cognition. In sum, information processing models are executed by the computational dynamics of neural circuits.

Two distinct modes of malfunction can occur when circuit dynamics execute models of information processing. The processing models describing behavior may fail to be executed correctly by neural mechanisms, without the physiological mechanism itself malfunctioning. Or, the neural mechanisms may malfunction, thereby failing to implement the right computation. To illustrate this view, we will assess two examples of psychiatric deficits, one each from a model and a mechanism malfunction. As an example of malfunctioning models, purported failures of rule following can be understood as failures to appropriately execute a suite of processing models. As an example of malfunctioning mechanisms, maladaptive behavior resulting from dysfunction in the mPFC can be understood as failures in the signal transformations carried out therein. The purpose of these examples is not to be definitive but rather descriptive, to illustrate our approach to neuropsychiatric nosology and the potential benefits it holds for the diagnosis and etiology of neuropsychiatric illness and dysfunction.

## Model-based dysfunction: failures to follow rules

A fundamental executive function in cognitive systems is the ability to follow rules. Two prominent clinical assessments of rule-following are the Iowa Gambling Task (IGT) (Bechara, 2007) and the Wisconsin Card Sort Task (WCST) (Milner, 1963). Both tasks have been proposed to assess the ability of patients to appropriately incorporate information to augment future behavior (“rule following”). We contend that these tasks probe many cognitive functions at once, thus failing to probe a unique cognitive function, due to each task enlisting multiple cognitive functions for successful performance.

The IGT (Bechara et al., 1994; Bechara, 2007) is a risk-based gambling task. Subjects are given an initial sum of money and attempt to maximize their profit by selecting cards from one of four decks. The different decks pay out randomly, with some decks advantageous over the long term, resulting in a net gain, and others not, resulting in a net loss. The IGT is utilized to assess cognitive function in patients with focal brain lesions, as well as a number of psychiatric illnesses (Bechara, 2007). Patients with ventromedial prefrontal cortex (vmPFC) lesions exhibit more disadvantageous deck choices (Anderson et al., 1999) and though they exhibit appropriate physiological responses to outcomes on the task, these patients show no anticipatory affective autonomic response prior to selecting from the risky decks (Bechara et al., 1996, 1999). There is also a laterality effect, where right vmPFC damage results in impairment and left vmPFC damage does not, hypothesized to be connected to the association of right hemispheric activity with affective processing (Tranel et al., 2002; Clark et al., 2003; Buelow and Suhr, 2009). Some studies find that dorsolateral prefrontal cortex (dlPFC) and dorsomedial prefrontal cortex (dmPFC) damage results in deficient choices compared to controls (Manes et al., 2002; Fellows and Farah, 2005), while others fail to find such deficits in dlPFC lesioned patients (Bechara et al., 1998; Bechara and Damasio, 2002; Bechara, 2003; Fellows, 2004). Patients with amygdalar lesions show a deficit in IGT performance (Bechara et al., 1999; Brand et al., 2007a,b), though such patients exhibit general physiological deficits. Adding to this confusion, neuroimaging studies of healthy controls implicate medial orbitofrontal cortex (mOFC) in mediating IGT performance (Ernst et al., 2002; Bolla et al., 2003; Tucker et al., 2004; Windmann et al., 2006) (for review, see Buelow and Suhr, 2009).

Although there has been criticism of the IGT in the past (Buelow and Suhr, 2009; Gansler et al., 2011a,b), the IGT is a perfect illustration of the shortcomings of focusing on gross anatomical and behavioral deficit. What formal processing must the patient's brain execute in order to behave optimally on the task? Patients must track the values of each of the decks. Tracking this value requires risk encoding, encoding of the variance of rewards relative to the mean reward (Weber et al., 2004; McCoy and Platt, 2005). Risk and value computations potentially explain the mOFC activation present in neuroimaging during the IGT, as the mOFC encodes the value of options in the environment and mediates reward-guided decision making (Padoa-Schioppa and Assad, 2006; Noonan et al., 2010; Walton et al., 2010; Watson and Platt, 2012). In addition to value and risk, optimal performance on the IGT requires tracking reward rates over time, a fundamental capacity of organisms that is aptly captured by optimal foraging theory (Stephens and Krebs, 1986). Dopaminergic signals originating in the basal ganglia (BG) play a fundamental role in reward signaling (Schultz et al., 1997, 1998), subjects with dopaminergic disorders such as Parkinson's patients show deficits in foraging behavior (Rutledge et al., 2009), and dopaminergic pathways originating in the BG innervate the PFC (Williams and Goldman-Rakic, 1993; Björklund and Dunnett, 2007). Disorders evident in IGT behavior may result from a failure to execute the computations described by foraging models, particularly resulting from deficits in the targets of dopaminergic projections to prefrontal areas. Risk assessment, expected value computations, reward intake rates, and presumably other cognitive processes are implicated in successful IGT performance. Thus, the IGT assesses behavior in a fashion that combines multiple cognitive functions.

Analyzing evidence from the WCST suggests a similar conclusion. Utilized in the assessment of frontal lobe dysfunction, the WCST requires the subject to match a sample card with one of four key cards along one of three dimensions: color, number and shape (Grant and Berg, 1948; Milner, 1963; Heaton, 1981, 1993). The subject is not informed of the matching rule, but receives feedback after each sample card about whether the categorization was correct. After 10 consecutive correct matches, an unsignaled change in the active matching rule occurs, and the subject has to explore to determine the new rule. The subject's performance can be analyzed along a number of dimensions, including the number of completed rule switches, number of perseverative errors (sticking with an old rule after a switch), and number of non-perseverative errors (switching from a correct rule) (Nyhus and Barceló, 2009). A number of brain regions have been implicated in successful WCST performance. Milner's original 1963 study found more perseverative errors for dlPFC lesioned patients than those with OFC, temporal, or parietal lesions (Milner, 1963). The relative importance of frontal cortex for WCST has since been corroborated by a large number of studies (see Nyhus and Barceló, 2009 for recent review). However, damage to temporal (Hermann et al., 1988; Corcoran and Upton, 1993; Strauss et al., 1993; Horner et al., 1996; Giovagnoli, 2001), subcortical (Mukhopadhyay et al., 2007), hippocampal (Corcoran and Upton, 1993; Giovagnoli, 2001; Igarashi et al., 2002), and cerebellar (Mukhopadhyay et al., 2007) regions impairs WCST performance. Neuroimaging studies of patients or normal controls have linked increased activation of the dlPFC (Kawasaki et al., 1993; Marenco et al., 1993; Berman et al., 1995; Nagahama et al., 1996, 1997; Volz et al., 1997; Mentzel et al., 1998; Nagahama et al., 1998; Ragland et al., 1998; Lombardi et al., 1999; Rogers et al., 2000; Monchi et al., 2001; Wang et al., 2001; González-Hernández et al., 2002; Lie et al., 2006) and vlPFC (Monchi et al., 2001; Lie et al., 2006), among other, non-frontal areas (Nyhus and Barceló, 2009), to successful behavior on the task.

The breadth and number of regions activated suggests that the WCST requires a number of different cognitive functions for successful performance. For example, “set-shifting,” the ability to switch between active rules, is often invoked as one of the main functions probed by the WCST and disturbed in patient populations that exhibit WCST deficits (Barceló et al., 1997; Rubinstein et al., 2001; Monsell, 2003; Braver et al., 2006; Shallice, 2006; Nyhus and Barceló, 2009). The notion of set-shifting as switching between encoded rules may be an outdated legacy of the classical approach to theorizing about how rules are executed (Haugeland, 1985). Instead, as with the IGT, optimal performance on the task may be driven by cognitive foraging mechanisms as subjects search through an abstract space of possible patterns of behavior to determine the adaptive response (Hills et al., 2008), which requires keeping track of reward rates or constructing and updating models of the environment. In particular, upon a decrease in the local reward rate, the system may be forced into an exploratory regime where it attempts to determine the current rule. Individuals with nonspecific brain injury have been shown to exhibit deficits on the WCST and a foraging task, exhibiting a preference for local reward rates (Schlund, 2002), indicating the potential benefits of viewing aberrant WCST behavior through the lens of formal foraging theory. Similar lessons may be gleaned by reconceptualizing how errors on the WCST are classified and analyzed. Non-perseverative errors have been criticized in the past as conflating two types of error, called efficient (appropriately exploring for a new rule) and random (inappropriately switching rules) errors (Barceló and Knight, 2002). Perhaps, however, the neural mechanisms underlying WCST performance evolved to support exploratory search, where changes in behavior permit exploration for and potential exploitation of new resources. If so, both types of error are forms of exploratory behavior owing to foraging through possible behavioral patterns in order to optimize reward rates. Likewise, perseverative errors, associated with dlPFC lesions (Rogers, 1998; Shallice, 2006), may not result from failures to disengage from previously activated rules, but failures to appropriately assess reward rates or failures to integrate local reward rates with information about the environment, both resulting in failures to forage in the space of possible actions to maximize reward. Much like the IGT, the WCST assesses cognitive function in a fashion that may in fact cut orthogonally across multiple different cognitive computations needed for successful performance on the task.

## Mechanism-based dysfunction: malfunction in the medial prefrontal cortex

The mPFC consists of a group of cortical structures in the anterior end of the brain and includes the anterior cingulate cortex (ACC), the dmPFC, and the vmPFC, as well as other areas. The mPFC has been implicated in social cognition (Amodio and Frith, 2006), default mode activity (Buckner et al., 2008), cognitive control (Ridderinkhof et al., 2004), affective processing (Etkin et al., 2011), and other processes. Here we focus on the ACC and its role in implementing the mechanisms that subserve information processing in cognitive control and social cognition.

Recent research has revealed a stereotyped signal transformation in the ACC sulcus (ACCs) exhibiting integrate-to-bound dynamics and that may be instructive for psychiatric pathologies like obsessive-compulsive disorder (OCD). Integrate-to-bound dynamics in neuronal mechanisms implement a basic exponentiation function that starts at a baseline, integrates some incoming signal until reaching a threshold, and then resets (Usher and McClelland, 2001; Wang, 2002). This basic circuit has been implicated in a diverse array of functions from eye movement control (Andersen, 1989; Seung, 1996, 2003) to perceptual decision-making across modalities (Hernandez et al., 2002; Roitman and Shadlen, 2002; Uchida and Mainen, 2003; Uka and DeAngelis, 2003, 2006; Heekeren et al., 2004; Romo et al., 2004; Kepecs et al., 2006; Uchida et al., 2006). Recently Hayden and colleagues unveiled in ACC a novel implementation of the integrate-to-bound circuit over longer timescales for strategic decisions (Hayden et al., 2011). Monkeys participated in a simulated foraging task, deciding when to leave a steadily decreasing reward source to forage at another, with leave decisions penalized by a timeout, simulating travel to a new rewarding location. As monkeys neared a decision to leave, neurons in the dorsal and ventral banks of the ACCs exhibited a pattern of successively higher peaks of activation, with this activity thresholding on leave decisions (Hayden et al., 2011). This activation pattern, observed in the past for sequences of movements (Shidara and Richmond, 2002), is highly suggestive of an exponential signal transformation by the integrate-to-bound dynamics in ACCs neurons.

The regularity of the activation function of neurons in this area has implications for the role of ACC in neuropsychiatric dysfunction, as illustrated by the case of OCD. OCD has a well-characterized clinical pathology, affecting up to 3% of the population and resulting in anxiogenic intrusive thoughts (obsessions) and repetitive aversive actions (compulsions) (Graybiel and Rauch, 2000; Maia et al., 2008; Menzies et al., 2008; Nenadic, 2008; Greenberg et al., 2010). The aberrant neurobiological circuitry in OCD patients includes the head of the caudate (Guehl et al., 2008; Maia et al., 2008) and orbitofrontal cortices (Maia et al., 2008; Menzies et al., 2008), as well as the anterior cingulate (Saxena et al., 1998; Cosgrove and Rauch, 2003; Maia et al., 2008). All three areas show hyperactivity in functional neuroimaging at rest (Szeszko et al., 2008), increased activation with OCD symptom provocation (Saxena et al., 1998), and resolution of resting state hyperactivity with successful therapy (Maia et al., 2008). The ACC specifically shows elevated levels of baseline activity in OCD (Rauch et al., 1994, 2001; Adler et al., 2000) and hyperactivation during cognitive activity such as during the processing of errors (Fitzgerald et al., 2005) or during incongruent trials in a conflict task (Fitzgerald et al., 2005, 2010; Maltby et al., 2005; Page et al., 2009). Cingulotomy, which resects both banks of the ACCs and the ACC gyrus (ACCg), is effective as treatment for refractory OCD (Graybiel and Rauch, 2000; Cosgrove and Rauch, 2003; Kim et al., 2003; Greenberg et al., 2010; Dougherty and Greenberg, 2011).

What role in the dysfunctional signal processing in OCD does the ACC play? ACC multiplexes information about rewards and actions (Hayden and Platt, 2010), and lesions in monkeys to the ACCs result in difficulty in reward-guided changes in behavior (Kennerley et al., 2006). Removal of both ACCs and ACCg results in higher error rates in a reward harvesting task (Rushworth et al., 2003). In humans, single neuron activity in ACCs in OCD patients has been shown to signal trials where different possible responses interfere as well as elevated activity following these interference trials, and the concomitant behavioral slowing following an interference trial was abolished post-ACC resection (Sheth et al., 2012). But the mechanism approach reveals deeper insight into the signal transformations that are central to the function of the ACC.

As the ACCs signals changes in behavioral strategy or sequence, a deviant signal transform in the form of persistent transient activation that thresholds abnormally may result in both an inability to change an action plan—compulsions—as well as the hypermetabolism observed in the ACC in OCD patients. The dysfunction in ACCs can be helpfully analyzed using dynamical systems theory. In dynamical systems theory, a system is conceptualized as inhabiting a point in its state space, the space of all possible states of the system. As the system evolves over time, it occupies a succession of such states in its state space, tracing out a trajectory of such states. Some states in the state space will attract the system; these are known as attractors, and the states around the attractor that typically lead to the system occupying that state are known as the basin of attraction for that attractor. OCD symptoms could reflect an inability of the circuit to leave a basin of attraction resulting in a repeated pattern of activation that does not threshold as normal. This hypothesis is supported by modeling of OCD with dynamical systems, which suggests that attractor basins in the network are abnormally deep, compelling the system to stay in a particular basin, possibly due to either excitatory glutamatergic hyperactivity (Rolls et al., 2008) or decreased inhibition (Maia and McClelland, 2012). As the system's signal transforms become trapped in a pattern of repeated, non-increasing transient activations, the behavioral output likewise becomes trapped in a pattern of repeated actions, the compulsions that are a hallmark of OCD.

Knowledge of the neural dynamics in a particular brain region connects metabolic evidence with the computations being executed in a region for tailored interventions. Granted knowledge of the dynamics implemented by an anatomical circuit, its hypo- or hyper-activity can be correlated with aspects of the signal transformation being performed by the circuit that might be malfunctioning in a particular disorder. The circuit mechanism approach allows us to characterize the ways that local circuits transform signals such that intervention might be tailored to suit the malfunctioning transform (Tass, 2003). Treatments such as deep brain stimulation promise to be more effective if they can be targeted to the specific dysfunction in the signal transformation carried out by the local malfunctioning circuit (Nenadic, 2008). Understanding the local signal transformations executed by neuronal mechanisms also allows tailored resection for therapeutic intervention in refractory psychopathology; for example, instead of removing the whole ACCs and ACCg, as has been done in the past (Graybiel and Rauch, 2000; Sheth et al., 2012), focal resection of the ACCs may be sufficient. Likewise, tailored deep brain stimulation could be designed to boost the system out of a local basin of attraction by injecting current at precise times. Understanding what signals are transformed, how they are transformed, and where they are transformed allows for both tailored interventions and bespoke resections.

Other encoding processes in ACC provide insight into the signal transformations relevant to disorders of social cognition. Reference frame encoding and coordinate transformations are fundamental computational functions for executing cognitive functions (Pouget and Snyder, 2000; Andersen and Buneo, 2002; Cohen and Andersen, 2002). In reference frame encoding, neural populations encode behaviorally relevant variables in a reference frame, a coordinate system oriented along some dimension, such as an egocentric dimension like eye position or arm position, or an allocentric dimension, like the position of the target in the world. Each neuron in a population exhibits a preference for a specific value of the relevant variable, and the population's response encodes the variable's value (Glimcher and Sparks, 1992). In the sensorimotor transform from sensation to action, signals encoded in one or more sensory dimensions must be transformed via synaptic computations and efferent signal transmission into the effector-appropriate motor dimensions. This process of reference frame encoding, signal transformation and transmission is a basic organizational feature of information processing in the brain (Poggio, 1990). The reference frame transformation mechanism is just one of the types of computations implemented by neural circuit dynamics to execute cognitive functions, and dysfunction in ACC in certain disorders can be interpreted as disorders of reference frame encoding.

Extending the reference frame concept to reward processing, we propose that deficits of social cognition can result from deficits in reward reference frame encoding. Reward reference frames are different for different prefrontal areas, as revealed by a recent study investigating reward encoding in rhesus monkeys choosing whether or not to donate juice rewards to other monkeys (Chang et al., 2013). In this study, reward was delivered to the actor subject, to another non-actor subject in the room with the actor, or to neither. Recordings from OFC, ACCs, and ACCg revealed that OFC cells preferentially encoded received rewards, ACCs cells preferentially encoded foregone rewards, and ACCg neurons were mixed, with neurons showing preference for received rewards, donated rewards, and for both donated and received rewards, indicating a mixed egocentric/allocentric reference frame encoding. This mixed encoding is a hallmark of areas using intermediate level transformations, those part way in transforming sensory signals into motor ones, in the sensorimotor mapping necessary for adaptive action (Pouget and Snyder, 2000; Chang, 2013).

The reference frame framework provides a number of new hypotheses about the origin of social deficits in atypical cognition. Social cognitive functions are a species of cognitive function and, like other cognitive computational processes, are executed using the basic computational building blocks of dynamic signal transformations. The ACCg has been implicated in deficits in following social gaze cues (Vecera and Rizzo, 2004, 2006), a fundamental social capacity of primates (Shepherd, 2010), and lesions in rhesus macaques have demonstrated that the ACCg is central to social cognition (Rudebeck et al., 2006). Mechanistic malfunction in executing social cognitive functions such as occurs in autism may occur in the encoding reference frame itself, such as problems in tuning or in the trajectory traversed through the state space of the population (Amit, 1992). It may also occur in the coordinate transformations necessary for adaptive social cognition, such as problems in the intermediate layer's implementation of functions necessary for coordinate transformations (Chang, 2013). In any case, failures of social competence may result from failures to implement the appropriate processing, the reference frames, and coordinate transformations requisite for social cognition.

## Conclusion

As illustrated in the foregoing, neuropsychiatric evaluation often focuses on deficits evident in particular tasks or on characterization of anatomical insult (Damasio and Damasio, 1989). But research in cognitive neuroscience has unveiled two levels, the model and the mechanism, and neuropsychiatric illness can manifest as dysfunction at either of these levels. Model dysfunction results from failures to execute the right information processing model for adaptive behavior at a mechanism level, and multiple such functions may be disrupted on a given task, calling for care in the design and interpretation of behavioral assays for mental illness. Mechanism dysfunction results from failures to implement the right signal transformations at a neurophysiological level, resulting in maladaptive behavior and psychiatric illness. Models and mechanisms are tightly wound together, as the execution of a model requires the implementation of the right mechanisms.

We have elsewhere urged scientists and clinicians to note that neural circuits malfunction as a result of state-shifted or variance-shifted processing (Chang et al., 2012). The addition of noise into a circuit yields variance-shifted dysfunctions, resulting in suboptimal and noisy processing, while shorting or destroying a circuit yields state-shifted dysfunctions, resulting in ablated or radically atypical behavior (Chang et al., 2012). Applying this distinction in types of malfunction to models and mechanisms, cognitive dysfunction could result from a state-shifted or variance-shifted model or from a state-shifted or variance-shifted mechanism. A state-shifted model is simply the execution of the wrong or incorrect model for a particular behavior. A variance-shifted model is the execution of the correct model, but with the model variables being encoded in a noisier-than-typical fashion. Likewise, a state-shifted mechanism is one whose dynamics are no longer qualitatively identical to those prior to insult, and a variance-shifted mechanism is one whose dynamics are qualitatively identical but with increased variability. When considering this cognitive function space as a whole, each disorder will carve out some dysfunction subspace.

While every executed model requires a suite of mechanisms, models and mechanisms can fail independently. The failure of a mechanism need not result in a failure to execute a model due to redundancy. And just like death by a thousand cuts, aggregation of variance-shifted mechanisms can result in a state-shifted model. Furthermore, executing a computational model requires the joint operation of multiple mechanisms. If this joint operation is disrupted, such as by severing the connections between mechanisms, then despite the preserved functioning of the component mechanisms, the model may still fail to be executed properly, whether ablated altogether (as in a state-shifted dysfunction) or executed in a noisy fashion (variance-shifted dysfunction).

Cognitive neuroscience increasingly invokes models of information and signal processing to explain cognitive phenomena. This neurocomputational approach to psychiatry agrees well with other, recent proposals for understanding the computational basis of psychiatric dysfunction, such as defining new computational and cognitive phenotypes for classifying individuals (Maia and Frank, 2011; Montague et al., 2012; Robbins et al., 2012). By defining the dynamic space of cognitive neural activity, classes of individuals may travel through this space in distinct fashions that correspond to disorders or symptoms, but that may also yield novel classifications orthogonal to preexisting analyses of mental illness. We urge practitioners to take note of this possibly revolutionary transformation in the way that neuroscientists are explaining neurocognitive function, as it provides a framework for the analysis, interpretation and treatment of psychiatric dysfunction.

And as for Vladimir, who exhibited such maladaptive behavior? His persistent rule following may have been the result of an inability to properly follow directions, or of an inability to properly assess the execution of a rule and transition to a new behavior (Goldberg, 2001). Note, though, that he was not completely incapable of following directions, only of executing certain sorts of control over his behavior, such as preventing an old instruction from informing current behavior or inhibiting an action plan once initiated, redolent of the types of rule following deficits observed on the WCST or of the sorts of compulsions seen in OCD patients. Due to the extreme damage he suffered, the absence of control structures based on foraging models, dysfunctional reinforcement learning or belief updating, or other types of damaged information processing may better account for his dysfunctional behavior than appeals to classic, qualitatively described functions such as rule following, rule assessment, or rule transitions.

## Author contributions

All authors listed have made substantial, direct and intellectual contribution to the work and approved it for publication.

### Conflict of interest statement

The authors declare that the research was conducted in the absence of any commercial or financial relationships that could be construed as a potential conflict of interest.
